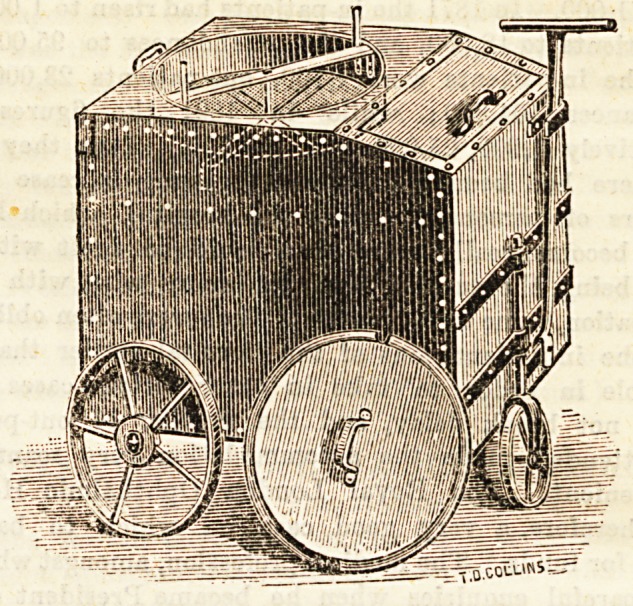# Cinder-Bin and Sifter

**Published:** 1892-05-28

**Authors:** 


### CINDER-BIN AND SIFTER.

A contrivance more especially likely to prove useful to large households and institutions has been invented by Messrs. Cook in the form of a combined cinder-bin and sifter, of which we give an illustration below. It has been found most satisfactory at several workhouses and other large establishments. By the adoption of this contrivance the dust-hole is altogether done away with. It may remain in any convenient position adjacent to any building perfectly stationary, by the handle being turned at right angles and secured in a small hook attached for the purpose, as shown in the drawing:

By raising the top round lid the cinders may be placed in the sifter, and by a few circulatory movements, which are quite noiseless, with the handles attached, the cinders are completely sifted, and can then be removed with perfect ease. When the bin is sufficiently full it may be wheeled off the premises into the road by the dustman, and there emptied of its contents by lifting the sliding back, thereby saving a great amount of labour hitherto incurred. The accumulation of rubbish under the old system is both disagreeable and unhealthy, but by the adoption of this simple machine the nuisance is effectively abolished; its advantages both hygienic and economic are obvious. The cinder-bin and sifter is strongly made with angle iron corners, and galvanized after made. The prices are from £3 15s. to £5 15s., according to size.

## Figures and Tables

**Figure f1:**